# Real-Time PCR-Based Analysis of the Human Bile MicroRNAome Identifies *miR-9* as a Potential Diagnostic Biomarker for Biliary Tract Cancer

**DOI:** 10.1371/journal.pone.0023584

**Published:** 2011-08-17

**Authors:** Kengo Shigehara, Shigeki Yokomuro, Osamu Ishibashi, Yoshiaki Mizuguchi, Yasuo Arima, Yutaka Kawahigashi, Tomohiro Kanda, Ichiro Akagi, Takashi Tajiri, Hiroshi Yoshida, Toshihiro Takizawa, Eiji Uchida

**Affiliations:** 1 Department of Surgery for Organ Function and Biological Regulation, Nippon Medical School, Tokyo, Japan; 2 Department of Molecular Medicine and Anatomy, Nippon Medical School, Tokyo, Japan; Istituto Dermopatico dell'Immacolata, Italy

## Abstract

Biliary tract cancer (BTC) is often difficult to diagnose definitively, even through histological examination. MicroRNAs (miRNAs) regulate a variety of physiological processes. In recent years, it has been suggested that profiles for circulating miRNAs, as well as those for tissue miRNAs, have the potential to be used as diagnostic biomarkers for cancer. The aim of this study was to confirm the existence of miRNAs in human bile and to assess their potential as clinical biomarkers for BTC. We sampled bile from patients who underwent biliary drainage for biliary diseases such as BTC and choledocholithiasis. PCR-based miRNA detection and miRNA cloning were performed to identify bile miRNAs. Using high-throughput real-time PCR-based miRNA microarrays, the expression profiles of 667 miRNAs were compared in patients with malignant disease (*n* = 9) and age-matched patients with the benign disease choledocholithiasis (*n* = 9). We subsequently characterized bile miRNAs in terms of stability and localization. Through cloning and using PCR methods, we confirmed that miRNAs exist in bile. Differential analysis of bile miRNAs demonstrated that 10 of the 667 miRNAs were significantly more highly expressed in the malignant group than in the benign group at *P*<0.0005. Setting the specificity threshold to 100% showed that some miRNAs (*miR-9*, miR-302c*, *miR-199a-3p* and miR-222*) had a sensitivity level of 88.9%, and receiver-operating characteristic analysis demonstrated that *miR-9* and miR-145* could be useful diagnostic markers for BTC. Moreover, we verified the long-term stability of miRNAs in bile, a characteristic that makes them suitable for diagnostic use in clinical settings. We also confirmed that bile miRNAs are localized to the malignant/benign biliary epithelia. These findings suggest that bile miRNAs could be informative biomarkers for hepatobiliary disease and that some miRNAs, particularly *miR*-9, may be helpful in the diagnosis and clinical management of BTC.

## Introduction

Among the hepatobiliary malignant neoplasms, biliary tract cancer (BTC), which includes intra- and extrahepatic cholangiocarcinoma and gall bladder cancer [1], is increasing in incidence. BTC arises from the biliary tract epithelia and frequently causes stricture or obstruction of the bile duct [2]. A consequence of this obstruction is the retention of bile, which leads to jaundice. Patients suspected of suffering from BTC and presenting with clinical symptoms such as jaundice are usually examined using diagnostic imaging techniques including ultrasonography, computed tomography (CT), and magnetic resonance cholangiopancreatography (MRCP). However, these techniques have limited sensitivity for the detection of BTC [3], and establishing an accurate diagnosis of cancer in the clinical setting is often difficult because many benign disorders display similar symptoms. The result is often a delayed diagnosis of BTC [4]. Early detection of BTC is critical to patient outcome because a) BTC is associated with poor prognosis [4]; b) it is characterized by non-responsiveness/weaker response to chemotherapy and radiotherapy; and c) the only curative treatment is surgical resection [5], [6]. BTC patients often undergo surgery before their condition has been definitively determined to be malignant [7]. Thus, improvement in diagnostic accuracy for BTC is needed, particularly in light of the increasing global incidence of the disease. During the last 10 years, considerable progress has been made in understanding the pathogenesis of BTC, and several genes and proteins that contribute to the molecular mechanisms of biliary tract carcinogenesis have been identified [8], [9], [10], [11], [12].

MicroRNAs (miRNAs) are endogenously expressed, short, noncoding RNAs 18–25 nucleotides in length that repress protein translation by binding to target mRNAs. In the field of cancer, a number of studies have been conducted on tissue miRNAs in an effort to elucidate disease mechanisms and improve upon current diagnostic and prognostic indicators [13], [14], [15]. Recently, studies on the utility of miRNAs from extracellular components, notably serum and plasma, have been reported [16], [17], [18]. For example, it has been shown that placenta-associated circulating miRNAs correlate with pregnancy progression [16]. Cogswell *et al.* recovered miRNAs from cerebrospinal fluid and discovered Alzheimer's disease-specific miRNA changes consistent with their role as potential biomarkers for the disease [19]. Melkonyan *et al.* detected a variety of miRNAs in urine, including some excreted transrenally, and proposed new diagnostic possibilities for transrenal nucleic acid analysis [20]. Elsewhere, Michael *et al.* extracted miRNAs from exosomes obtained from the saliva of healthy controls and a patient with Sjögren's syndrome [21]. In the context of cancer, miRNAs in serum from patients with breast cancer and diffuse large-B-cell lymphoma have been shown to be stable and highly predictive of malignancy and survival [17], [18]. These reports suggest significant roles for extracellular miRNAs, in addition to those of tissue miRNAs, in regulating many physiological processes. As this field develops, these miRNAs may become diagnostic and therapeutic targets in clinically relevant situations. These reports promoted us to speculate that miRNAs may be present and stable in human bile, just as they are in other bodily fluids, and that bile-borne miRNAs could be used as novel biomarkers for BTC.

The major components of human bile are bile acid, cholesterol, bilirubin, bicarbonates, electrolytes, and water. Bile is produced by hepatocytes in the liver and flows via the bile duct into the duodenum, where it assists in lipid digestion and absorption [22]. Like blood, bile can be collected from patients who undergo diagnostic and/or therapeutic bile drainage. One can therefore understand the utility of a BTC biomarker in bile and/or serum. A bile biomarker could expedite the diagnosis of BTC by prompting further histological examinations such as bile cytology, brush cytology, and forceps biopsy of the bile duct.

In this study, we first examined whether miRNAs exist and can be detected in human bile by small RNA library sequencing. Next, we determined whether the expression of specific miRNAs in bile differs between patients with cancer and those without cancer using high-throughput real-time PCR-based miRNA expression microarrays. Finally, we assessed the potential of bile miRNAs as novel biomarkers for BTC.

## Materials and Methods

### Bile collection

The study protocol was approved by the Human Ethics Committee of Nippon Medical School and the Research Ethics Committee of Nippon Medical School. Patients who signed a written informed consent form were enrolled in this study. Bile samples were collected from 18 patients suffering from biliary disease (including BTC and choledocholithiasis) who underwent diagnostic and/or therapeutic bile drainage via percutaneous transhepatic cholangiodrainage, percutaneous transhepatic gall bladder drainage, or endoscopic nasobiliary drainage. Nine patients were diagnosed histologically with adenocarcinoma (seven with cholangiocarcinoma and two with gall bladder cancer). We included nine age-matched control patients diagnosed with choledocholithiasis without current or previous malignancy or an inflammatory condition (**Table 1**).

**Table 1:** Patient characteristics.

Malignant (*n* = 9)						
No.	Diagnosis	Pathology	Sex	Age	CEA(ng/ml)	CA19-9(U/ml)
M1	Cholangiocarcinoma	Adenocarcinoma	M	49	1.5	70.8
M2	Cholangiocarcinoma	Adenocarcinoma	F	78	1.6	35.8
M3	Cholangiocarcinoma	Adenocarcinoma	M	68	2.9	485.5
M4	Cholangiocarcinoma	Adenocarcinoma	F	74	1.4	33.1
M5	Gallbladder cancer	Adenocarcinoma	F	82	7.5	105.7
M6	Gallbladder cancer	Adenocarcinoma	F	55	0.5	6.1
M7	Cholangiocarcinoma	Adenocarcinoma	F	71	0.8	51.2
M8	Cholangiocarcinoma	Adenocarcinoma	F	78	1.5	0.8
M9	Cholangiocarcinoma	Adenocarcinoma	M	59	2.3	65

Benign (*n* = 9)						
No.	Diagnosis	Pathology	Sex	Age	CEA(ng/ml)	CA19-9(U/ml)
B1	Choledocholithiasis	N.A.	F	62	W.N.L.	
B2	Choledocholithiasis	N.A.	M	70	W.N.L.	
B3	Choledocholithiasis	N.A.	M	91	W.N.L.	
B4	Choledocholithiasis	N.A.	F	54	W.N.L.	
B5	Choledocholithiasis	N.A.	M	64	W.N.L.	
B6	Choledocholithiasis	N.A.	M	50	W.N.L.	
B7	Choledocholithiasis	N.A.	M	45	W.N.L.	
B8	Choledocholithiasis	N.A.	M	80	W.N.L.	
B9	Choledocholithiasis	N.A.	M	64	W.N.L.	

CEA, carcinoembryonic antigen (normal range <2.5 ng/ml); CA 19-9, carbohydrate 19-9 (normal range <37 U/ml); N.A., not applicable; W.N.L., within normal limits.

### Sample processing and RNA extraction

RNA was extracted from 5 ml of bile from each individual on the same day as collection. The nucleic acid fraction was obtained by extraction in phenol–chloroform–isoamyl alcohol (25∶24∶1) followed by ethanol precipitation (−80°C for 30 min). Total RNA was obtained using RNAiso Plus (TaKaRa Bio, Tokyo, Japan) according to the manufacture's protocol. Extracted RNA samples were stored at −80°C until use.

### Real-time PCR

Total RNA was reverse-transcribed using non-specific primers/primers specific for each mRNA/miRNA, respectively. Triplicate samples of the resultant cDNA were subjected to real-time PCR using TaqMan miRNA primers/probes (Applied Biosystems, Tokyo, Japan) with a 7300 Real-Time PCR System (Applied Biosystems).

### Small RNA library sequencing of bile miRNAs

miRNA cloning methods are described in our previous report [23]. Briefly, total RNA samples were extracted from bile using RNAiso Plus (TaKaRa Bio) (see above) and separated in a denaturing polyacrylamide gel. The 18–24-nt fraction was then recovered. Next, 5′- and 3′-adapters were ligated to the RNAs, and real-time PCR was carried out. Amplification of the cDNA fragments was achieved through two consecutive rounds of PCR. Specific restriction enzyme digestion of the adaptors allowed for concatemerization of the cDNA into larger fragments. These fragments were then cloned into a vector to create a cDNA library. Concatemerization increases the lengths of the informative sequences that may be obtained from each clone. The small RNA sequences were analyzed for homology with known miRNAs. Annotation of the miRNAs was performed using the miRBase (latest version) homology analysis function.

### Real-time PCR-based miRNA expression profiling

Total miRNA (350 ng) was reverse-transcribed using Megaplex RT Primers (Applied Biosystems). The resulting cDNAs were pre-amplified using Megaplex PreAmp Primers (Applied Biosystems) and the pre-amplified products applied to a TaqMan Human MicroRNA Array Panel (A and B, v2.0). Real-time PCR was performed using a 7900HT Fast Real-Time System (Applied Biosystems). The real-time PCR data obtained using the TaqMan MicroRNA Panel were analyzed with Applied Biosystems software (SDS ver. 2.3 and RQ manager ver. 1.2). miRNAs expression levels for patient M9 (see **Table 1**) were set to 1.0. miRNAs whose expression was not detectable in patient M9 (see **Table 1**) were excluded from further analysis. For quantification, the relative CT method (ΔΔCT method) was applied. U6 was used as an internal control. For all 18 patients, this assay was performed using Panels A and B. We determined the cut-off for significance using a tuning parameter delta, chosen on the basis of a false-positive rate of zero. The array data were used to construct receiver-operating characteristic (ROC) curves by plotting the relationships between true positives (sensitivity) and false positives (1 – specificity). The area under the curve (AUC), a measure of discrimination accuracy, was also calculated.

### miRNA stability assay

To evaluate the stability of endogenous miRNAs in bile, we measured miRNA levels in bile from three individuals that had been incubated at room temperature for 0 to 24 h. Briefly, 5 ml of each bile sample was incubated, and miRNAs were extracted and purified at the scheduled time points using the method described above. RNA samples were then reverse-transcribed and subjected to real-time PCR. To measure the stability of exogenous miRNA, a synthetic *Caenorhabditis elegans* miRNA (*cel-miR-39*) was added to each bile sample at the start of the incubation period (0 h) and subjected to the same extraction and quantification procedures. To normalize the data, the synthetic *C. elegans* miRNA *cel-miR-238* was added to the nucleic acid fraction immediately prior to reverse transcription to serve as an internal control in the real-time PCR.

### Bile fractionation

**Figure 1:**
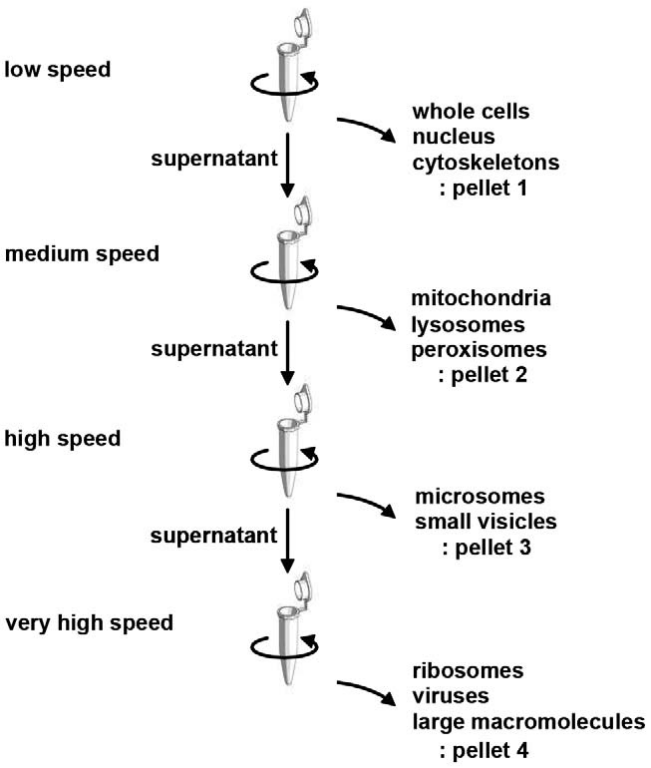
Differential centrifugation scheme for bile. Human bile was subjected to repeated centrifugation at progressively higher speeds to separate cells and organelles. The centrifugation conditions (gravity and time) were as follows: low speed, 1,000× g for 10 min; medium speed, 20,000× g for 20 min; high speed, 80,000× g for 1 h; very high speed, 150,000× g for 3 h.

Bile is reported to be fractionated into four components by increasing both the centrifugal force and the duration of centrifugation [24]. To assess bile miRNA localization, we fractionated 12-ml samples of bile from three individuals by differential centrifugation [25] and compared the expression level of the miRNAs in each fraction (**Fig. 1**). Relatively large components such as whole cells, nuclei, and cellular cytoskeletons sediment at a *low speed* (1,000× *g* for 10 min). Pellets containing mitochondria, lysosomes, and peroxisomes were obtained at a *medium speed* (20,000× *g* for 20 min), and microsomes and small vesicles at *high speed* (80,000× *g* for 1 h). Finally, the smallest components (e.g., ribosomes, viruses, and large macromolecules) were collected at a *very high speed* with a long period of centrifugation (150,000× *g* for 3 h) (**Fig. 1**). RNA was extracted from each pellet using RNAiso Plus (TaKaRa Bio) according to the manufacturer's protocol, and the expression of some arbitrarily selected miRNAs was measured by real-time PCR as described above. The expression level of the mRNA encoding the biliary epithelial marker cytokeratin 7 (CK-7) was also measured to verify that the fractionation had been conducted properly [26], [27], [28], [29].

## Results

### Confirmation of the existence of miRNAs in human bile

**Figure 2:**
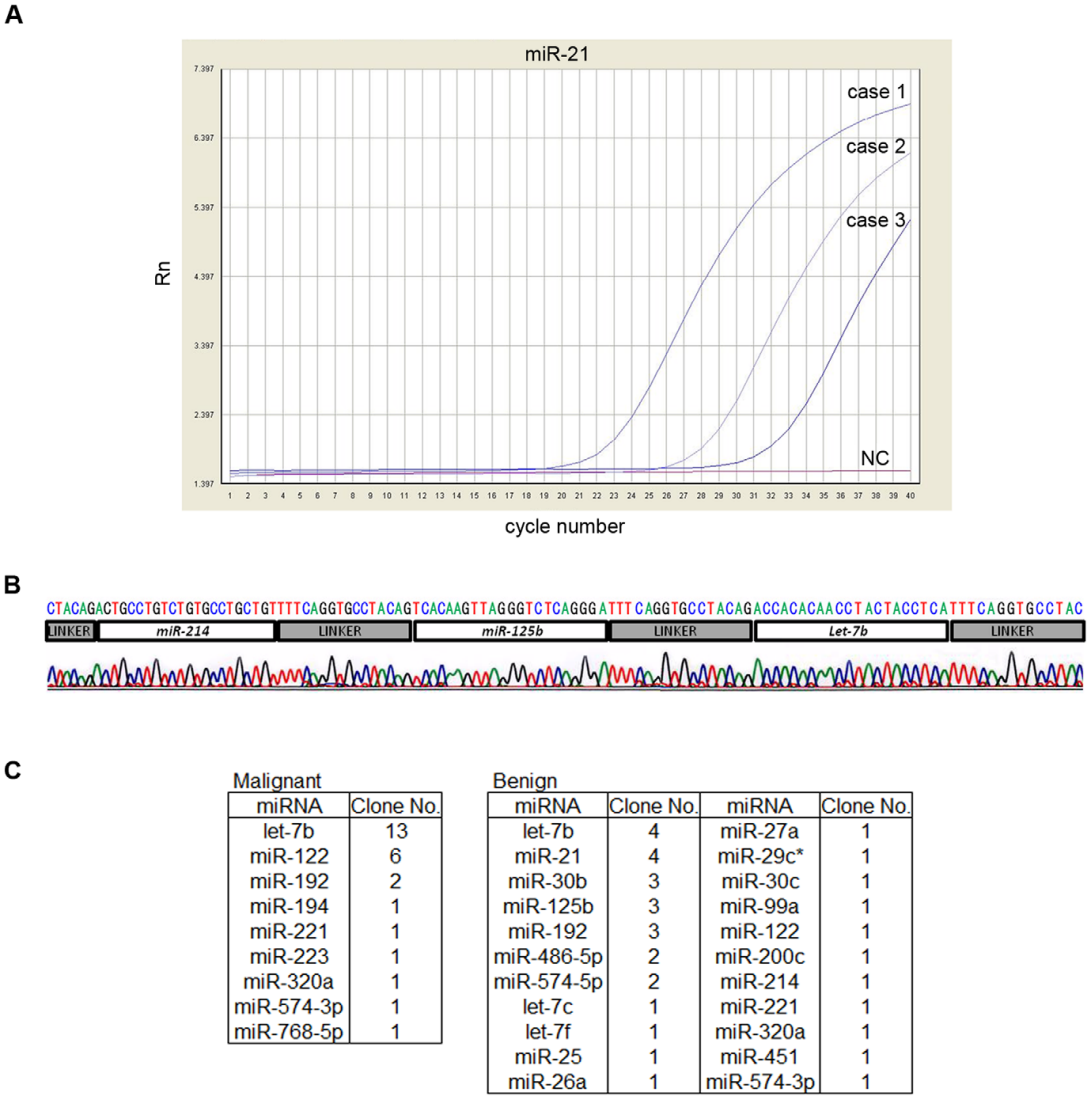
Confirmation of the existence of miRNAs in human bile. (A) Real-time PCR-based miRNA detection of *miR-21*. TaqMan miRNA real-time PCR using a probe/primers specific for *miR-21* was performed on three bile samples and a no-template control sample (NC). Note the lack of a PCR product in the negative control (NC). (B) Representative chromatogram of the sequencing results after cloning the bile miRNA. Horizontal bars above the chromatogram indicate each miRNA and linker (see Materials and Methods). (C) Table showing the miRNAs identified from cloning, their relative frequency, and whether they were cloned from BTC or benign cases.

To date, there has been no report on the presence of miRNAs in bile. Our first priority was to determine whether miRNAs exist in bile and, if so, to identify them. Real-time PCR with specific primers/probes demonstrated that *miR-21* was present in bile (**Fig. 2A**). Using bile as the source material, small RNA library sequencing verified the presence of several miRNAs, with *let-7b* being the most frequently cloned (**Fig. 2B and 2C**). These findings showed that miRNAs are present in bile and that bile is an attractive biological material for miRNA analysis.

### High-throughput and quantitative microRNA expression profiling of bile from patients with biliary tract cancer

**Figure 3:**
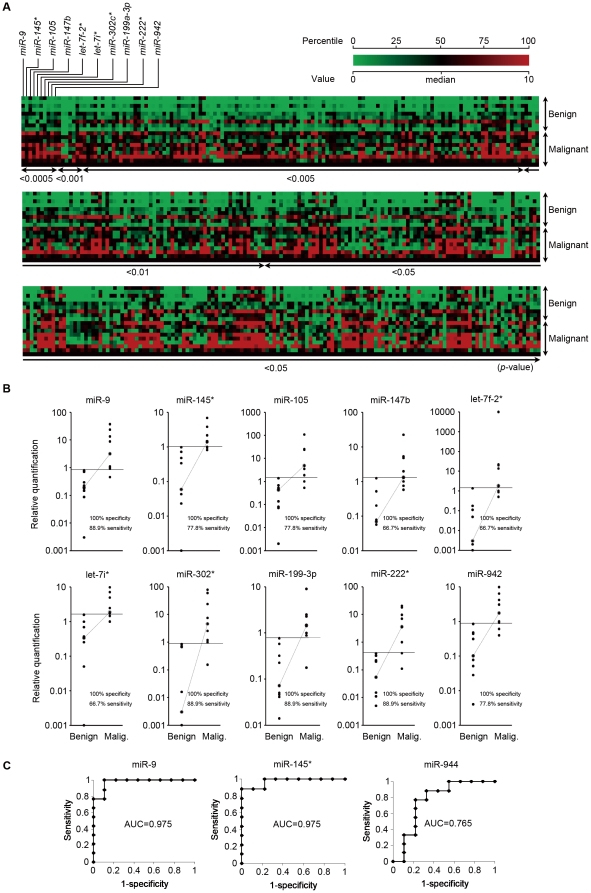
Comparison of malignant and benign samples using high-throughput real-time PCR-based miRNA microarrays. (A) miRNA expression heat map, with miRNAs sorted according to their *P*-values. Samples are shown in rows, and miRNAs in columns. miRNA expression in patient M9 (see **Table 1**) was set to 1.0. Complete microarray results can be found in the Supplementary Information. (B) Dot plot showing the expression levels of the 10 miRNAs that were significantly more highly expressed in the malignant group than in the benign group (*P*<0.0005). The vertical logarithmic axis shows relative quantification values obtained in a TaqMan array assay. Setting the specificity threshold to 100% showed the sensitivity level to be 88.9% for *miR-9*, miR-302c*, *miR-199a-3p*, and miR-222*; 77.8% in miR-145*, *miR-105*, and *miR-942*; and 66.7% in *miR-147b*, let-7f-2*, and let-7i*. ○, median value. (C) ROC curve analysis. The expression profile for each miRNA was used as the input for receiver operating characteristic (ROC) analysis. The ROC curve shows sensitivity versus 1 – specificity. The area under the curve (AUC), a measure of discrimination accuracy, was also calculated.

To identify bile miRNAs that are aberrantly expressed in biliary tract diseases, especially biliary tract cancers, we analyzed bile samples from all 18 patients by real-time PCR-based miRNA expression profiling (**Fig. 3A** and **Table S1**). Relative quantification of the data showed that 335 of the 667 miRNAs were significantly more highly expressed in the malignant group than in the benign group (*P*<0.05) (**Fig. 3A** and **Table S1**). Of these, we highlighted 10 miRNAs (*miR-9*, miR-145*, *miR-105*, *miR-147b*, let-7f-2*, let-7i*, miR-302c*, *miR-199a-3p*, miR-222* and *miR-942*) whose expression was significantly higher at *P*<0.0005 (**Fig. 3A**). Setting the specificity threshold to 100% [17], [30] showed that *miR-9*, miR-302c*, *miR-199a-3p*, and miR-222* had a sensitivity level of 88.9%, indicating that these miRNAs can serve as biological markers for biliary tract cancer (**Fig. 3B**). The sensitivity level for miR-145*, *miR-105*, and *miR-942* was 77.8%, and that for *miR-147b*, let-7f-2*, and let-7i* was 66.7% (**Fig. 3B**). Additionally, the area under the ROC-curve analysis showed that *miR-9* and miR-145* could be excellent diagnostic markers for BTC (**Fig. 3C**). Taken together, our data show that bile miRNAs, notably *miR-9*, have the potential to be used as diagnostic indicators for biliary tract cancers.

### Characterization of bile miRNAs

**Figure 4:**
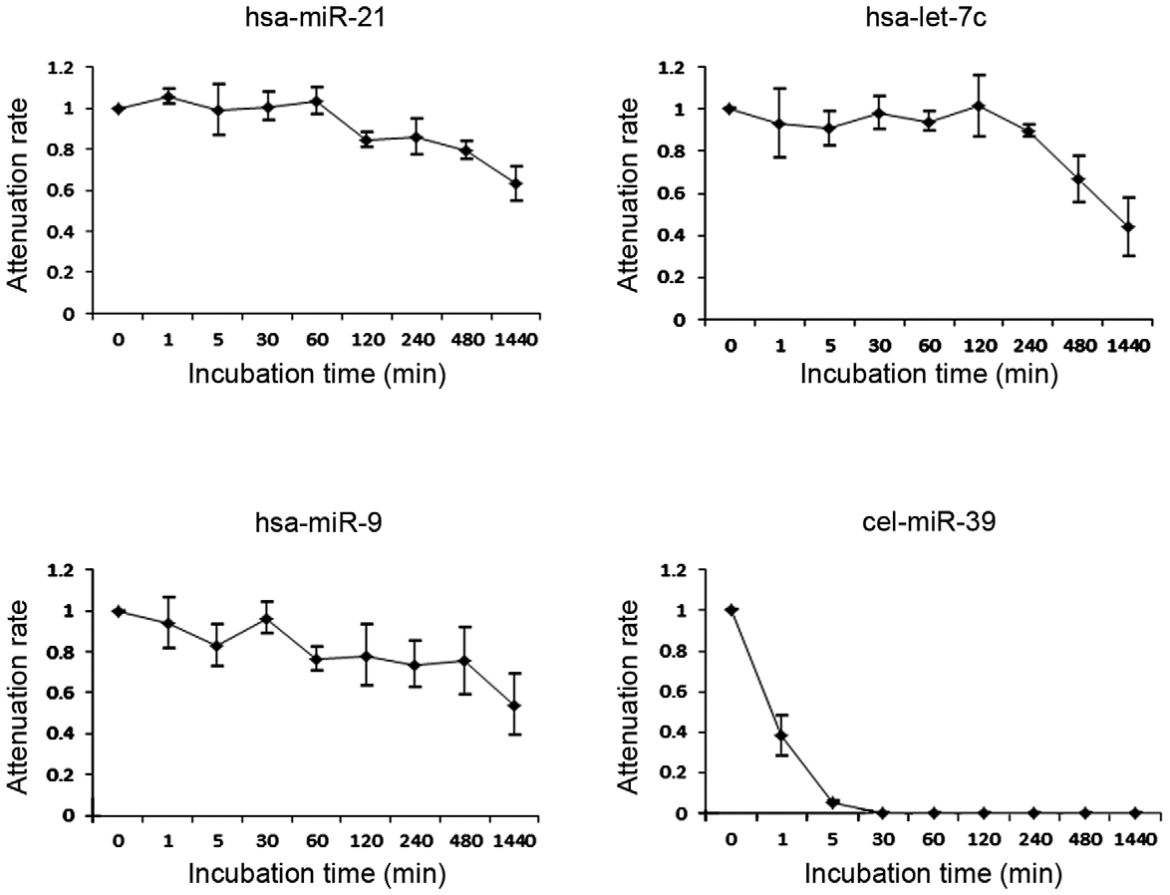
Time-dependent changes in endogenous and exogenous miRNAs in human bile. The endogenous human bile miRNAs *hsa-miR-21*, *hsa-let-7c*, and *hsa-miR-9* are relatively stable. In contrast, the synthetic exogenous miRNA *cel-miR-39* was rapidly degraded when added to bile.

**Figure 5:**
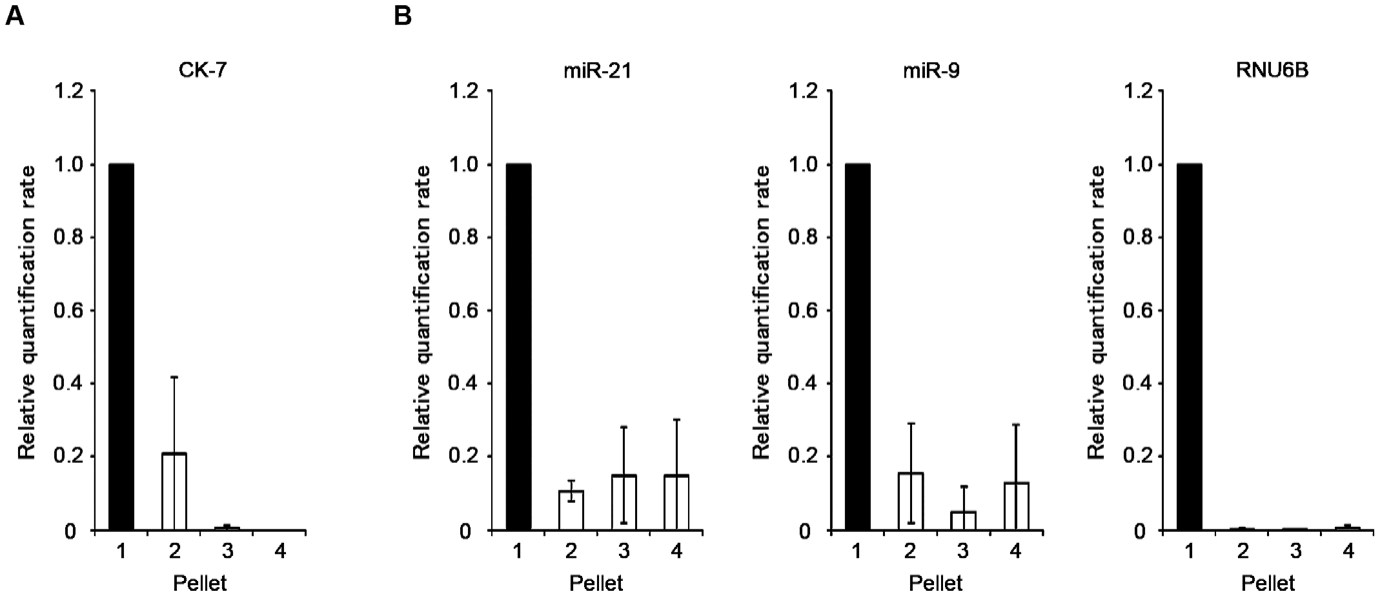
Relative quantification of CK-7 mRNA expression and miRNA levels s in each fraction of bile. Levels of mRNA and miRNAs in each fraction were calculated from the results of real-time PCR and the volume of total RNA to compare the simplified absolute quantity in each component. (A) mRNA encoding the biliary epithelium marker CK 7 was predominantly detected in the subcellular component, which included whole cells, nuclei, and cellular cytoskeletons. (B) The miRNAs *miR-21*, *miR-9*, and *RNU6B* showed a similar pattern, indicating that most miRNAs in bile were derived from biliary epithelial cells. Fractions were obtained through low-speed (1), medium-speed (2), high-speed (3); and very high-speed centrifugation (4).

For bile miRNAs to be used diagnostically in a clinical setting, they need to be stable. Considering its degradative nature, it was important to determine how long miRNA integrity could be maintained in bile. As shown in **Fig. 4**, we confirmed that the endogenous bile miRNAs *miR-21*, *let-7c*, and *miR-9* were stable in bile and that their expression levels remained high, especially within 4 hours of 24 hours incubation. However, the exogenous miRNA *cel-miR-238* was immediately degraded when added to the three individual bile samples (see Materials and Methods). These observations suggest that endogenous bile miRNAs are in a stable form that can withstand unfavorable conditions, such as potent RNase activity and strong acidity, that cause exogenous miRNAs to be degraded. Furthermore, the fractionation analyses showed that, for all three samples tested, all miRNAs, including *miR-9*, were detected in the component containing biliary epithelial cells, nuclei, and cytoskeletons, indicating that bile miRNAs reside primarily inside cells and nuclei. (**Fig. 5**).

## Discussion

Here, we report on the presence and stability of bile miRNAs, their relative expression levels, as measured by high-throughput real-time PCR, and differences in the miRNA profiles between bile samples from patients with benign and malignant biliary tract disease. After confirming the existence of miRNAs in bile using specific miRNA primers, we performed a more comprehensive analysis using miRNA microarrays, which allowed us to test for the presence of 667 miRNA species. Although this is a small study, we have confidence that our concept of using miRNAs to distinguish benign/malignant biliary tract diseases will be validated as more large-scale studies are conducted in the future. In addition to proposing the diagnostic use of bile miRNAs, we have developed reliable methods for extracting and evaluating miRNAs that are compatible with clinical testing. Comparative analysis of expression levels identified 10 miRNAs whose levels differ between malignant and benign conditions (*P*<0.0005) (**Fig. 3B**). ROC analysis identified two of these miRNAs as being suitable for use as BTC biomarkers (**Fig. 3C**). Notably, *miR-9* showed reliable diagnostic specificity and sensitivity.

In the context of cancer biology, aberrant expression of *miR-9* has been associated with metastasis [31], [32], [33], [34]. The *miR-9* family is upregulated [32], [33] or downregulated [31], [35] in various human cancers. The difference in *miR-9* expression seen here may be similar to the stage-specific regulation that was demonstrated for *miR-200* in liver cancer [36]. Early, when cancer cells become invasive and undergo epithelial–mesenchymal transition, *miR-200* is downregulated, but it is later upregulated during the reepithelialization of distal metastases when cells undergo mesenchymal–epithelial transition [36]. The mechanism of *miR-9* dysregulation in our study remains unknown but may involve aberrant methylation of is promoter region [31], [34], [37] or f activation of the transcription factor MYC/MYCN [32]. Further functional studies of *miR-9* in BTC are needed.

We subsequently investigated the biochemical characteristics of miRNAs in the bile. Plasma has been reported to show strong RNase activity [38]. Mitchell *et al.* confirmed this RNase activity and demonstrated that naked miRNAs were subject to degradation, whereas endogenous miRNAs remained stable in plasma, even in the presence of potent RNase activity [39]. Interestingly, we observed similar differences in stability between endogenous and exogenous miRNAs in bile (**Fig. 4**). The stability of endogenous bile miRNAs makes them suitable as reliable biomarkers in clinical situations.

How endogenous miRNAs withstand the denaturing conditions of bile has yet to be explained. Recent studies demonstrated that most of the circulating miRNAs are found in exosomes, which protect them from degradation and are responsible for their excellent stability [40], [41], [42]. Exosomes are 40–100-nm membrane vesicles of endocytic origin that contain both mRNA and miRNA. Exosomes can be transferred from one cell to another, and their components can function in the new environment. Several miRNA profiling studies have been conducted on exosomes circulating in the blood [40], [41], [42]. We initially presumed that the stability of endogenous bile miRNAs resulted from their presence in exosomes. However, contrary to our expectations, cell fractionation experiments revealed that most of the bile miRNAs were not found in small vesicles but in whole cells and nuclei (**Fig. 5**). Although there is no evidence that bile miRNAs have the same functions as those in serum or plasma, our findings from the fractionation studies and the PCR analysis of CK-7 suggest that miRNAs extracted from bile are mainly derived from intact epithelial cells or malignant cells that have been desquamated from the bile tract. Thus, endogenous miRNAs remain stable in the bile until degradation of the desquamated cells in which they are contained. Once miRNAs are released from these structures into the bile, they are likely rapidly degraded.

There is a real need for innovative tools to accurately diagnose BTC. Currently, the presence of carcinoembryonic antigen (CEA) and carbohydrate 19-9 (CA 19-9) in serum serve as a standard for the clinical diagnosis of BTC. As shown in **Table 1**, however, the sensitivity of this test is not as high as that achieved by our miRNA analysis. In eight of nine BTC cases, serum CEA levels did not increase, even though cancer was present. Additionally, serum CA-19-9 levels were only increased in certain patients, probably because of cholestasis. Diagnostic imagining techniques such as endoscopic ultrasonography (EUS) and intraductal ultrasonography are used when BTC is suspected. EUS allows for a good view of the distal extrahepatic biliary tree, gall bladder, regional lymph nodes and vasculature [5]. Rösch *et al.* compared the diagnostic accuracy of endoscopic retrograde cholangiopancreatography (ERCP), MRCP, CT, and EUS in 50 patients with biliary strictures. The sensitivity/specificity of diagnosis of malignancy was 85%/75% for ERCP, 85%/71% for MRCP, 77%/63% for CT, and 79%/62% for EUS [43]. In a prospective study of patients with suspected cholangiocarcinoma, EUS had a diagnostic sensitivity of 79% and a specificity of 62% [44]. Moreover, in a recent meta-analysis, EUS had a sensitivity of 78% and a specificity of 84% [45]. Intraductal ultrasonography with wire-guided, thin-caliber, high-frequency probes is performed during ERCP, and in previous studies showed accuracy rates for distinguishing benign and malignant strictures of 76–90% [46], [47], [48]. In addition to imaging techniques, histological methods such as bile cytology, brush cytology, and forceps biopsy are mandatory for definitive diagnosis. Bile cytology and brush cytology have only modest accuracy rates for determining malignancy, ranging from 30 to 70% in most published studies [5], [49], [50], [51], [52], [53]. Forceps biopsy, which has a higher accuracy rate than the other histological tests (43 to 81%), is not widely used because it requires a specialized device and technique [54], [55], [56]. Similarly, the more specialized EUS-guided fine-needle aspiration biopsy, with a diagnostic sensitivity of 43 to 86% for biliary strictures [7], [57], [58], [59], [60], [61], is currently only used for pancreatic tumors and inferior bile duct strictures and has not been approved for use in other conditions. The low diagnostic accuracy of some of the currently used tests confirms that BTC can be difficult to diagnose. In view of the poor prognosis for BTC [4], it is desirable to improve the ease and accuracy of testing. The results of our investigation indicate that measuring bile miRNAs can improve the speed and accuracy of diagnosing BTC. Furthermore, bile analysis is feasible because bile miRNAs are stable and can be easily extracted and analyzed in clinical settings.

Further investigations with larger numbers of patients and different study populations as well as studies of differential display between BTC and pre-malignant diseases such as primary sclerosing cholangitis are needed to achieve clinical acceptance. Nevertheless, our results demonstrate that measurement of bile miRNA levels is a practical approach for aiding the assessment of BTC and is comparable to many current diagnostic methods, including cytology. We therefore conclude that measurement of miRNA expression in bile would be helpful in distinguishing between benign and malignant conditions, especially in cases that remain undiagnosed. Notably, bile *miR-9* has strong potential for use as a clinical marker of biliary tract cancers.

## Supporting Information

Table S1 **Expression levels of the miRNAs in bile.** A heat map was constructed using data from the miRNA microarrays used to analyze bile samples from patients with malignant and benign disease. miRNA expression in patient M9 (see **Table 1**) was set to 1.0. B, analysis of bile from a patient with benign disease; M, analysis of bile from a patient with malignant disease.
(XLSX)
